# Left ventricular free wall impeding rupture in post-myocardial infarction period diagnosed by myocardial contrast echocardiography: Case report

**DOI:** 10.1186/1476-7120-4-7

**Published:** 2006-01-26

**Authors:** Maria Luciana Zacarias Hannouche da Trindade, Jeane Mike Tsutsui, Ana Clara Tude Rodrigues, Márcia Azevedo Caldas, José Antônio Franchini Ramires, Wilson Mathias

**Affiliations:** 1 Heart Institute (InCor) – University of São Paulo Medical School, São Paulo, Brazil

## Abstract

**Background:**

Left ventricular free wall rupture occurs in up to 10% of the in-hospital deaths following myocardial infarction. It is mainly associated with posterolateral myocardial infarction and its antemortem diagnosis is rarely made. Contrast echocardiography has been increasingly used for the evaluation of myocardial perfusion in patients with acute myocardial infarction, with important prognostic implications. In this case, we reported its use for the detection of a mechanical complication following myocardial infarction.

**Case presentation:**

A 50-year-old man with acute myocardial infarction in the lateral wall underwent myocardial contrast echocardiography for the evaluation of myocardial perfusion in the third day post-infarction. A perfusion defect was detected in lateral and inferior walls as well as the presence of contrast extrusion from the left ventricular cavity into the myocardium, forming a serpiginous duct extending from the endocardium to the epicardial region of the lateral wall, without communication with the pericardial space. Magnetic resonance imaging confirmed the diagnosis of impending rupture of the left ventricular free wall. While waiting for cardiac surgery, patient presented with cardiogenic shock and died. Anatomopathological findings were consistent with acute myocardial infarction in the lateral wall and a left ventricular free wall rupture at the infarct site.

**Conclusion:**

This case illustrates the early diagnosis of left ventricular free wall rupture by contrast echocardiography. Due to its ability to be performed at bedside this modality of imaging has the potential to identify this catastrophic condition in patients with acute myocardial infarction and help to treat these patients with emergent surgery.

## Background

Left ventricular free wall rupture occurs in up to 10% of the in-hospital deaths following acute myocardial infarction (AMI), usually between 3 to 6 days after the infarction. It typically involves the anterior or lateral wall, in the terminal region of the left anterior descending coronary artery distribution. It is associated with transmural infarctions involving at least 20% of the left ventricle, and it rarely occurs in areas with good collateral blood supply [1,2]. The local factors that lead to myocardial rupture are thinness of the apical wall at terminal end of blood supply, poor collateral flow and shearing effect of muscular contraction against an inert and stiffened necrotic area. Rupture of the left ventricular free wall usually leads to hemopericardium and death from cardiac tamponade. The course of rupture varies from a catastrophic event, with an acute tear leading to immediate death (acute rupture), or slow and incomplete tear leading to a late rupture (subacute rupture). Incomplete rupture may occur when the thrombus and haematoma together with the pericardium seal the rupture of the left ventricle and may develop into a diverticulum or a false aneurysm. The clinical recognition of rupture is often first suggested by development of profound right ventricular failure and shock progressing to electromechanical dissociation. Immediate pericardiocentesis will temporarily relieve tamponade followed by cardiopulmonary bypass and coronary artery bypass graft to repair wall. Although there is often insufficient time for diagnostic tests in patients in whom an acute left ventricular free wall rupture is suspected, echocardiography is the examination of choice. Echocardiography may demonstrate a pericardial effusion and typical findings of cardiac tamponade, however, identification of the rupture site is rarely possible. Contrast echocardiography, on the other hand, has the potential to diagnose free wall rupture prior to the development of tamponade [2,3]. Contrast medium can be visualized into the myocardium before reaching the pericardial space, suggesting an impending rupture. Additionally, direct extravasations of intravenous contrast agents into the pericardial space may be demonstrated in case of free wall rupture. In the present case we will demonstrate its usefulness in a patient with AMI.

## Case presentation

A 50-year-old man with history of systemic hypertension and cigarette smoking presented to the emergency room with epigastric pain that had begun two days earlier. Physical examination was unremarkable. Initial twelve-lead electrocardiogram displayed a 0.5 mm ST segment depression in leads V3 to V5, while cardiac markers showed elevated creatine kinase mass (32.8 ng/ml) and cardiac troponin (67.2 ng/ml). A diagnosis of a non-Q AMI was made. Because epigastric pain became worst, a new electrocardiogram was undertaken, revealing an 1 mm ST segment elevation in leads V2 to V5. Coronary angiography was then performed, which revealed 80% obstruction in left descendent anterior coronary artery, 90% obstruction in first diagonalis, 90% obstruction in right coronary artery and 90% obstruction in the second marginal branch of the circumflex coronary artery, with thrombus. Percutaneous transluminal coronary angioplasty was performed in this artery and resulted in TIMI flow II.

In the third day post infarction, the patient underwent a transthoracic echocardiogram for the evaluation of left ventricular systolic function. The patient was asymptomatic, blood pressure was 120 × 80 mm Hg, and there were no signs of hemodynamic instability. Transthoracic echocardiogram disclosed mild left ventricular hypertrophy, a moderately depressed left ventricular systolic function due to lateral wall akinesia and severe hypokinesia of the inferior and posterior walls, and a small pericardial effusion. The lateral wall was thin but no signs of impeding left ventricular rupture was detected by two-dimensional imaging. The patient gave informed consent to participate in a research protocol to assess post AMI perfusion by myocardial contrast echocardiography.

**Figure 1:**
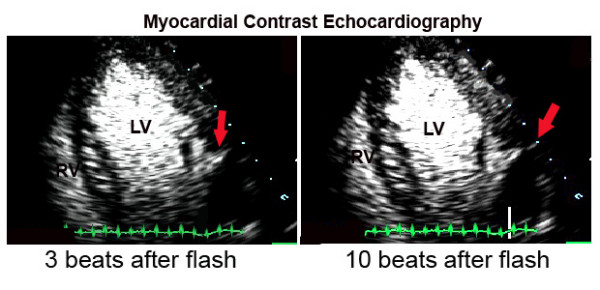
Myocardial contrast echocardiography images in the apical four-chamber view 3 beats after the high-mechanical index flash impulse (left panel) and 10 beats after the flash (right panel). The arrow shows the contrast extrusion from the left ventricular cavity into the myocardium in the lateral wall, before reaching the pericardial space, suggesting an impending rupture. LV = left ventricle; RV = right ventricle.

**Figure 2:**
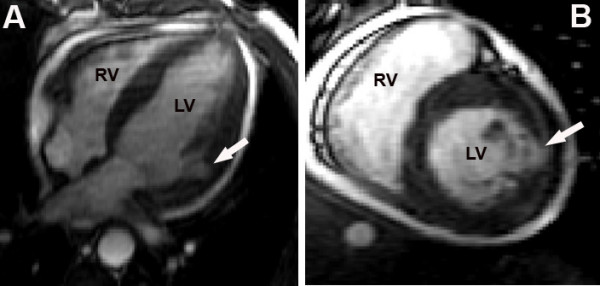
Magnetic resonance imaging in the apical four-chamber view (A) and short axis view (B). The arrows points to the presence of contrast in myocardium similar to that seen by contrast echocardiography. LV = left ventricle; RV = right ventricle.

**Figure 3:**
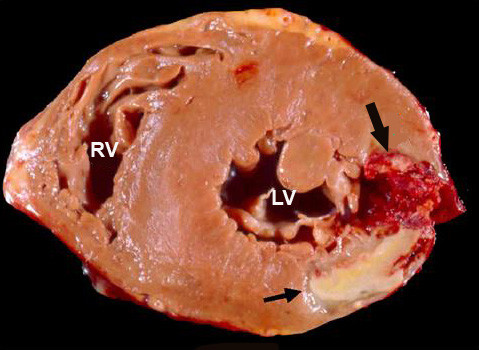
Anatomical slice of the left ventricle obtained during necropsy. The infarction region is between the arrows. The big arrow shows the region of left ventricular free wall rupture. LV = left ventricle; RV = right ventricle.

Myocardial contrast echocardiography was performed in the third day post-infarction with a commercially available system (SONOS 5500, Philips Medical Systems, Bothell, Washington, USA) equipped with a broadband (1–3 MHz) transducer (S3) and power modulation imaging, which is a pulse cancellation technique that deploys multiple pulses per image line, alternating full- and half-amplitude pulses [4]. The system was adjusted to achieve optimal non-linear signal at a mechanical index of 0.2, frame rate ≥ 25 Hz. To assess the replenishment kinetics, microbubble destruction in the myocardium was induced by manually-deflagrated flash containing five consecutive high-mechanical index (1.0) impulses. Sequences of low-power myocardial perfusion images containing at least 15 cardiac cycles after the flash were acquired in the apical four-, two- and three-chamber views. The contrast agent used was PESDA (Perfluorocarbon Exposed Sonicated Dextrose and Albumin), which is constituted by albumin-encapsulated microbubbles with mean concentration of 1.4 × 10^9 ^microbubbles/ml and mean size of 4.6 ± 0.2 microns [5]. A total of 0.1 ml/Kg of contrast agent was diluted in 80 ml of 0.9% saline and administered by continuous intravenous infusion. Real-time myocardial contrast echocardiography demonstrated a perfusion defect in the lateral and inferior walls. In addition, the presence of contrast extrusion from the left ventricular cavity into the myocardium could be observed, forming a serpiginous duct extending from the endocardium to the epicardial region of the lateral wall, without communication with the pericardial space (Figure 1). The hypothesis of an impending rupture of the left ventricular free wall was suggested, and the patient was taken to magnetic resonance within one hour. Magnetic resonance imaging demonstrated the same image of contrast extrusion into the myocardium (Figure 2), thus confirming the diagnosis. Urgent cardiac surgery was indicated. However, while waiting for surgery, the patient presented with cardiogenic shock followed by cardiorespiratory arrest. An immediate pericadiocentesis was performed, but it was followed by the patient's death in spite of resuscitation maneuvers. Anatomopathologic findings were consistent with AMI in the lateral wall and a left ventricular free wall rupture at the infarct site (Figure 3), corresponding to the area seen by contrast echocardiography.

## Discussion

The current indication of contrast agents in echocardiography includes left ventricular opacification and endocardial border delineation [6]. However, myocardial contrast echocardiography has been increasingly used in patients with AMI for the evaluation of myocardial perfusion. The presence of no-reflow after AMI is associated with irreversible microvascular damage and has been shown to predict left ventricular remodelling [7]. In addition, some reports in the literature have described its use for the evaluation of cardiac rupture. Mittle et al [2] presented two cases in which contrast echocardiography was able to exclude rupture in one case and helped identify a pseudo aneurysm in the other. Waggoner et al [3] illustrated the use of intravenous injections of a contrast agent during two-dimensional echocardiography in a patient with myocardial rupture after AMI. Besides contrast echocardiography, live three-dimensional echocardiography has also been reported to show accurate images of the actual rupture site [8]. However, it has not been shown to detect it before the ventricle has actually ruptured. Recently, non-invasive diagnosis of an impending left ventricular free-wall rupture was also reported by magnetic resonance [9].

The assessment of myocardial perfusion by contrast echocardiography has been performed by different techniques including high mechanical index triggered imaging, in which destruction of microbubbles is decreased by intermittent imaging triggered with cardiac cycles, and real-time imaging [4]. Real-time imaging utilizes a low-mechanical index that causes minimal microbubble destruction, and permits the simultaneous evaluation of myocardial perfusion and enhanced endocardial border definition following the administration of intravenous ultrasound contrast agents [10]. The use of this modality of imaging in this particular case permitted the demonstration of absence of myocardial perfusion in the lateral and inferior wall, as well as the identification of the discontinuity in the left ventricular myocardium. Our findings were confirmed by magnetic resonance imaging. Since the prognosis of a free wall rupture is very poor, even when surgical therapy is contemplated [1], we could speculate that patients might fare better if early diagnosis and management of this frequently fatal condition were performed. Although in our case emergent surgery could not be performed in properly time to avoid patient death, we would like to emphasize that the diagnosis was performed before the actual rupture occurred.

## Conclusion

Contrast echocardiography seems to be an alternative non-invasive method for the early diagnosis of left ventricular free wall rupture. Due to its ability to be performed at bedside, this modality of imaging has the potential to identify this catastrophic condition following acute myocardial infarction and help to treat these patients with emergent surgery.

## Competing interests

The author(s) declare that they have no competing interests.

## Authors' contributions

MLZHT and MAC acquired the echocardiographic images and participated in the manuscript description. ACTR, JMT and JAFR prepared the manuscript and figures. WMJ participated in the design and review of the manuscript. All authors read and approved the final manuscript.
